# Importance of Spring Habitats for Amphibians: The Case of Estavelle Ecotones in the Classical Karst Region

**DOI:** 10.3390/ani15223228

**Published:** 2025-11-07

**Authors:** Damiano Brognoli, Elia Lo Parrino, Giorgia Terraneo, Giorgio Grassi, Veronica Zampieri, Matteo Galbiati, Valentina Balestra, Valeria Messina, Benedetta Barzaghi, Stefano Lapadula, Raoul Manenti

**Affiliations:** 1 Dipartimento di Scienze e Politiche Ambientali, Università degli Studi di Milano, Via Celoria, 10, 20133 Milano, Italy; damianobrognoli00@gmail.com (D.B.); elia.loparrino@unimi.it (E.L.P.); terraneogiorgia002@gmail.com (G.T.); giorgio140199@gmail.com (G.G.); verozampieri@gmail.com (V.Z.); matteo.galbiati1995@gmail.com (M.G.); valeria.messina@unimi.it (V.M.); benedetta.barzaghi@unimi.it (B.B.); stefano.lapadula@unimi.it (S.L.); 2 Department of Environment, Land and Infrastructure Engineering, Politecnico di Torino, Corso Duca degli Abruzzi, 24, 10129 Torino, Italy; valentina.balestra@hotmail.com; 3 Biologia Sotterranea Piemonte—Gruppo di Ricerca, Loc. Bossea, 10 (Grotta di Bossea), 12082 Frabosa Soprana, Italy; 4 Laboratorio di Biologia Sotterranea “Enrico Pezzoli”, Parco Regionale del Monte Barro, Loc. Eremo, 1, 23851 Galbiate, Italy

**Keywords:** source, cave, pond, salamander, toad, frog, freshwater, fish, *Proteus*

## Abstract

**Simple Summary:**

This study explores how amphibians use springs, especially estavelles, in the Classical Karst Region. Estavelles are springs that can also act as sinkholes or ponors when water levels drop. Despite their importance, little is known about amphibian use of these habitats. The research surveyed 61 springs from June 2020 to January 2025, recording amphibian presence, breeding stages, and environmental factors such as water flow, habitat features, and predators. Results showed that over two-thirds of springs exhibited estavelle behavior at least once. Five amphibian species were identified, with some regularly found in springs for movement and breeding, while others used them temporarily. Amphibian presence was strongly linked to springs with low water flow, habitat complexity, and absence of predators like pike. The findings suggest that estavelles and surrounding areas serve as important shelters and feeding sites, especially for frogs. Overall, the study highlights the ecological importance of estavelles for amphibian survival, providing valuable insights for the study and conservation of neglected animals in karst landscapes.

**Abstract:**

Springs are ecotones between groundwater and surface water, important for a variety of both surficial and subterranean organisms. However, their use by amphibians has been poorly assessed. This is evident considering estavelles, typical ecotones functioning not only as spring but also as sinkholes. Here we aim to assess the use of estavelles by amphibians in the Classical Karst Region. From June 2020 to January 2025, we surveyed 61 springs, both during day and night. We visually recorded the occurrence of amphibians, along with abiotic and biotic features—including the presence of pikes (*Esox cisalpinus*), water flow level, drying events, and substratum heterogeneity. Forty-three springs exhibited estavelle-like hydrological behavior at least once. We identified five amphibian species. The use of springs was frequent for *Proteus anguinus*, *Rana latastei,* and *Pelophylax* sp. Amphibians’ occurrence was significantly linked to non-perennial estavelles characterized by low water flow, habitat heterogeneity, and absence of pike. Overall, reproductive activity in estavelles was limited; breeding likely occurs in nearby damp lentic habitats connected to the springs. Our results provide the first herpetological assessment of estavelle spring habitats in the Classical Karst Region, emphasizing their role as shelters for epigean species and feeding patches for stygobionts.

## 1. Introduction

Ecotones are dynamic habitats that host and shape a remarkable diversity of animal species [1,2,3]. They can occur across a broad spectrum of habitats, being transitional environments where distinct ecological features and pressures converge and interact [4,5,6]. Springs and resurgences well exemplify ecotones, being the boundaries between markedly different habitats such as groundwater and surface freshwater environments. Springs are predicted to attract a wide variety of organisms and involve complex ecological processes [7,8,9,10,11]. Furthermore, springs can provide valuable insights into the primary patterns driving evolutionary and ecological responses to contemporary challenges, including global warming [5]. Springs and their neighboring surface habitats can be more or less a marginal part of the distribution within an aquifer for various animal species that are usually considered as strictly adapted to groundwater [12,13,14,15,16]. These animals are defined as stygobionts (meaning inhabitants of the Styx, the subterranean river of Greek mythology) and often show typical traits like absence of eyes, depigmentation, low metabolic requirements, etc.) [17,18]. Stygobionts in springs can interact with typical surface animals like macroinvertebrates, fish, and amphibians [14,19,20,21].

Amphibians, which are themselves generally characterized by an important transition phase in their life cycle, such as metamorphosis, can play an important ecological role in spring habitats. The existence of distinct developmental stages enables amphibians to mount partially independent responses to the various selective pressures encountered across different habitats throughout their lifespan [22,23]. Nonetheless, the influence of environmental conditions experienced during early life stages rarely disappears entirely when the organism transitions to adulthood [24,25,26,27,28]. For instance, the biotic and abiotic factors encountered during the larval phase of amphibians can significantly shape the metamorphosis process, as well as influence traits and fitness outcomes after metamorphosis [29,30]. Amphibians in turn can affect the features of springs through exchanges of biomass and, subsequently, trophic energy across the groundwater/freshwater environment and the surrounding terrestrial habitats, as observed in many other contexts [31,32,33]. During breeding and aquatic phases, adult amphibians and their eggs (or egg-clutches) can provide subsidization to spring habitats [33,34] and, vice versa, with metamorphosis they can subsidize the terrestrial environment with resources acquired during the aquatic phase [34,35]. Despite the use of various ecotones by amphibians being investigated multiple times in recent decades [1,2,36,37], a limited number of studies investigates their use of spring habitats [16,19,38,39,40].

In karst areas, the so called “estavelles”, a particular type of spring/freshwater ecotones, are quite common [41,42,43,44]. They are interfaces/orifices between surface and underground environments that, depending on the weather conditions and season, can serve either as a sink or as a source of fresh water [45,46,47,48]. Depending on their position in the karst system, they can even become desiccated completely during dry periods [45,49,50]. Estavelles can likely provide valuable habitats. Thanks to their close connection to groundwater, estavelles can provide freshwater habitats with a relatively stable microclimate. Temperatures can be colder than surrounding waters in summer, as reported for other spring habitats [51,52,53]. Moreover, estavelles can increase environmental diversity in the whole karst ecosystem. Variations in the hydroperiod can strongly affect amphibian occurrence. From a side, breeding can be disadvantaged because larval development can require long periods of water occurrence. On the other side, estavelles can provide shelters for metamorphosed amphibians, especially if they are not strongly connected to larger water bodies, because fish and other predators can be absent.

Recent studies underlined that estavelles can be used by groundwater-dwelling amphibians like the olm *P. anguinus* [54]. This species is an obligate paedomorphic and stygobiont salamander, historically considered to occur in surface freshwater only when passively drifted out from groundwater [55]. However, the active exploitation of surface sites like estavelles seems quite common in the Classical Karst Region [56]. It is likely that *P. anguinus* emerges in estavelles and other spring habitats for preying upon both aquatic and terrestrial invertebrates [54]. Moreover, typical surface-dwelling amphibians like frogs, newts, and toads are likely to exploit estavelles, which, depending on their hydroperiod, can theoretically provide favorable breeding sites.

The aim of this study is to investigate the exploitation of estavelles and springs in karst habitats by amphibians, characterize this exploitation, and assess which features can favor it.

## 2. Materials and Methods

### 2.1. Study Area

**Figure 1:**
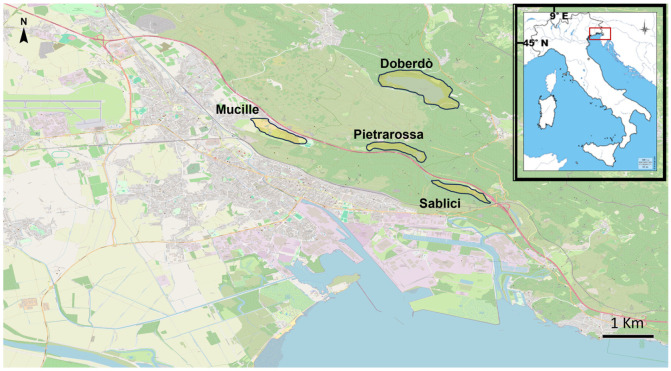
Map of the study area. Light yellow areas represent the location of the four poljes investigated.

The study area occurs within the western part of the Classical Karst Region, Italy, characterized by the occurrence of four karst poljes/lakes between Doberdò del Lago and Monfalcone municipalities (Figure 1). This area is rich with estavelles and karst springs in the surrounding of the so called “Lacus Timavi”, an extended damp area known since Roman times [57], nowadays mostly replaced by industrial and other anthropic buildings. From a herpetological point of view, the study area is relevant, hosting different amphibian species [58], including the Italian agile frog (*Rana latastei*) and the Italian crested newt (*Triturus carnifex*), with important conservation and ecological value [59,60,61,62].

### 2.2. Surveys

From June 2020 to January 2025 we performed repeated surveys in all the months of the year, August excluded, both during day and night, in 61 spring sites. We considered springs occurring in the four poljes that were likely to have the typical hydrological regime already described for estavelles. Springs were chosen following suggestions of local researchers, using past reports [63,64], or detected directly during field explorations. In total we performed 1224 observations with an average (±SE) number of visits per spring of 20.06 ± 1.88.

At each site, amphibian occurrence and breeding were assessed visually by inspecting all potential shelters, including substrate elements, for at least 10 min. Breeding was recorded when eggs, egg-clutches, larvae/tadpoles, or mating adults were detected. The presence of metamorphosed individuals (juveniles and adults) was also noted. Occasionally, some individuals were captured with a dip net and handled gently and briefly for identification. Such handling was authorized by the Italian Ministry of Environment and Energy Security (permits: 6957, 13 February 2020; 26,340, 3 March 2022), notwithstanding Council Directive 92/43/EEC.

At each survey, for each spring, we recorded abiotic and biotic features that can be important in explaining amphibians’ use of aquatic sites. As abiotic features we recorded the maximum water depth, the intensity of water flow, whether the site was emitting or absorbing, the morphology of the spring mouth, and diversification of substrate, which can reflect the abundance of shelters. Water flow was differentiated in four categories by the same observer: 1 = absent, 2 = just occurring, 3 = moderate, and 4 = strong. Diversification of substrate was based on microhabitat heterogeneity, considering the percentage cover of sediments (including organic matter) which was classified as follows: 1, no diversification, one single substrate element covering almost entirely the site; 2, poor diversification, occurrence of two different substrates that together covered >90% of the spring; 3, quite diversified, at least three elements on the substrate, each of which covering 20–40% of the spring habitat; and 4, highly diversified, four or more elements including leaves and branches. As biotic features we recorded the occurrence of aquatic and semi-aquatic macrophytes and the occurrence of individuals (both juveniles and adults) of the pike (*Esox cisalpinus*) which is the top predatory fish of the system investigated [65].

### 2.3. Statistical Analyses

We used a constrained redundancy analysis (RDA) to evaluate the relative role of spring features on the amphibians’ species composition. RDA allows evaluation of how much of the variation in the structure of one dataset (e.g., amphibian community composition in springs; endogenous dataset) is explained by independent variables (e.g., spring abiotic and biotic features; exogenous datasets) [66]. We built a matrix of environmental features considering the whole study period and composed it by the following: (1) the maximum water flow that we recorded; (2) if the spring dried up at least once during the whole study period; (3) the maximum depth reached by springs; (4) occurrence (or not) of aquatic macrophytes; (5) maximum heterogeneity of the microhabitat; (6) if the spring showed at least once during the whole study period the hydrological features typical of an estavelle; and (7) if we recorded pike occurrence at least once.

We used as an endogenous matrix, the species composition considering those for which we had enough records, such as the occurrence of *P. anguinus* (both adults and juveniles), adults of green frogs (*Pelophylax* sp.), *Bufo bufo*, and *R. latastei*. A species was considered present if it was detected at least once during the entire study period. We used variance partitioning to calculate the independent and joint effects of spring features, while the significance of the explained variance was assessed by 10,000 ANOVA-like permutation tests. RDA was performed using ‘rda’ and ‘permutation’ functions of the package ‘*vegan*’ [67,68] in R 4.5.1

## 3. Results

During four and a half years of fieldwork, we observed that 43 out of the 61 surveyed springs exhibited at least once the characteristic hydrological behavior of estavelles with water flow alternating between emission and absorption and thus functioning as both sources and sinks. The remaining sites were either perennial or ephemeral rheocrene springs or perennial limnocrene springs. Rheocrene springs are sources that feed lotic freshwater environments [69]. In our study area, rheocrene springs fed small watercourses that converge towards the middle of the polje. Limnocrene springs typically feed lentic freshwater sites [69,70]; in our study area, they supplied large perennial pools within the main poljes. Notably, fourteen springs did not display the typical “spring cup” at their outlets; instead, they consisted of small interstices and crevices of varying sizes. Ten springs presented a conical shape at the entrance, while thirty had a “spring cup” forming a pool. The remaining seven sites were fissures of at least 10 cm that were directly connected with groundwater.

**Figure 2:**
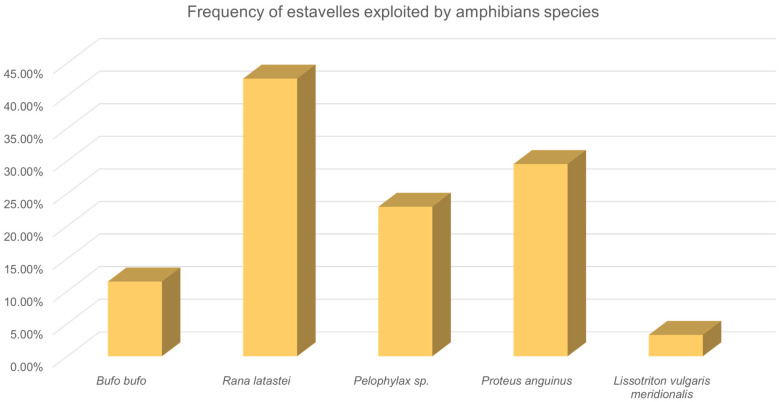
Percentage of the surveyed springs exploited by the different species of amphibians recorded. The graphs show the cumulative occurrence of the species including egg-clutches, tadpoles/larvae, and metamorphosed individuals.

We detected five taxa of amphibians, such as the Italian agile frog (*Rana latastei*), the olm (*Proteus anguinus*), the green frog (*Pelophylax* sp.), the smooth newt (*Lissotriton vulgaris meridionalis*), and the common toad (*Bufo bufo*). We also observed the Italian crested newt (*Triturus carnifex*) but only in adjacent sites of estavelles and springs and never directly connected with them (Figure 2).

Breeding was rarely observed in the estavelles monitored. It occurred regularly only for *R. latastei* in one site, a large perennial spring pool, where multiple egg-clutches were observed during each spring of the survey. Egg-clutches of the same species were also observed in an ephemeral estavelle once. Tadpoles of *B. bufo* and *R. latastei* were observed in six and three sites, respectively, while larvae of *L. v. meridionalis* and *P. anguinus* were observed in one estavelle, but likely their breeding occurred in different places.

**Figure 3:**
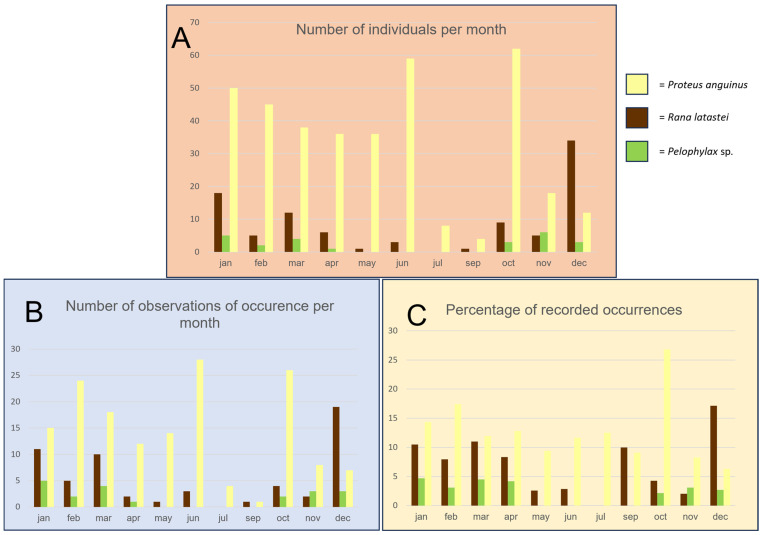
Months of observation of the three more regular species of amphibians detected, such as the olm (*Proteus anguinus*) showed in light yellow, the Italian agile frog (*Rana latastei*) showed in dark brown, and the green frog (*Pelophylax* sp.) showed in green, during the four years of surveys (June 2020–January 2025). (**A**) number of individuals of the three species observed. (**B**) number of occurrences at each spring during each month. (**C**) percentage of occurrences considering the whole number of surveys performed each month in each spring.

Considering the whole study period, the species recorded more regularly were *R. latastei*, *P. anguinus,* and *Pelophylax* sp. (Figure 3). The maximum number of individuals of *R. latastei* was observed in the December months of the four years of monitoring, while for *P. anguinus*, 62 individuals were recorded in June (Figure 3A). The number of individuals and observations of the green frogs was quite limited and occurred only in spring and autumn months (Figure 3A,B). Considering the relative percentage of records with respect to the total number of observations collected across the different months of the four-year monitoring period, observations of R. latastei peaked in winter and autumn months, while those of *P. anguinus*, although more regular, peaked in June and February (Figure 3C).

**Figure 4:**
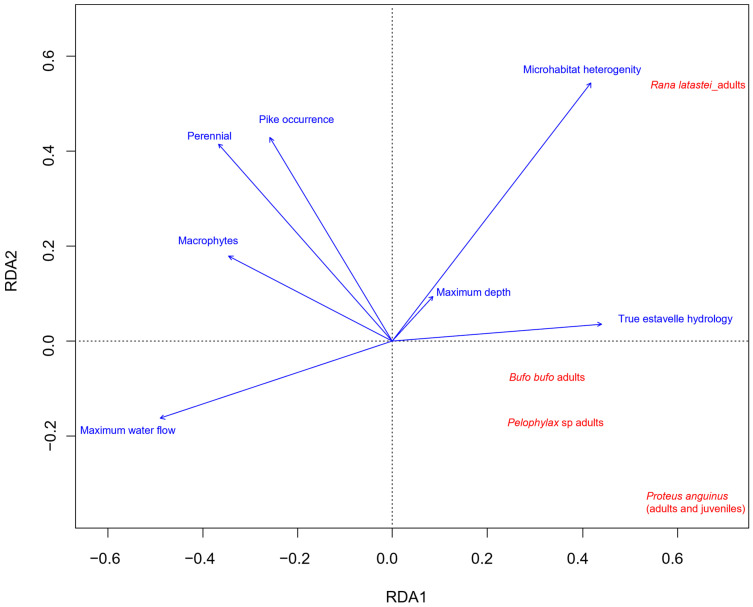
Results of the constrained redundancy analysis (RDA) between amphibians (red) and different environmental features of springs (blue).

Amphibians were significantly associated with the features of estavelles (F = 2.74; *p* < 0.01). The recorded environmental variables explained 26.61% of the variation. The first RDA axis was primarily related to perennial springs characterized by low maximum water flow, typical hydrological features of estavelles, high microhabitat heterogeneity, and absence of macrophytes. The second RDA axis was associated with perennial springs with pikes and high heterogeneity of the microhabitat (Figure 4).

All amphibian species showed a positive correlation with the first RDA axis and a negative correlation with the second, except for *R. latastei*, which was positively associated with both axes (Figure 4). *R. latastei* appears strongly linked to springs with high microhabitat heterogeneity and typical estavelle hydrology. *P. anguinus* was clearly associated with estavelles and temporary springs lacking predators (Figure 4).

**Figure 5:**
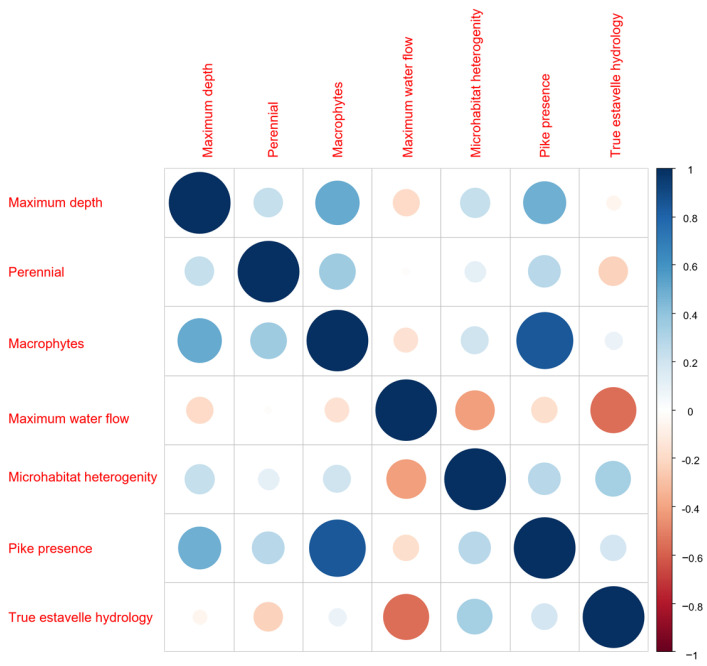
Correlogram of the variables considered to describe estavelles’ features.

A correlation analysis considering the variables in the exogenous matrix showed that estavelles with pike presence are associated with greater maximum depth and the presence of aquatic macrophytes (Figure 5). In addition, maximum water flow was negatively correlated with microhabitat heterogeneity (Figure 5).

## 4. Discussion

Our study on amphibian use of karst springs and estavelles in the Classical Karst Region provides new insights into the ecological role of these freshwater ecotones. We documented six amphibian species in total. The Italian crested newt was recorded only incidentally, and the smooth newt and the common toad were observed only occasionally. By contrast, three additional species were recorded relatively regularly, frequently exploiting estavelles for foraging, reproduction, or refuge. These findings highlight the heterogeneous use of karst ecotones by amphibians and underscore the conservation value of estavelles as complementary aquatic habitats in the karst landscape.

Notably, one of these three species is the olm (*Proteus anguinus*), which is a stygobiont salamander, only recently studied even in surface freshwater habitats [56,71]. Even if *P. anguinus* individuals from Classical Karst Region, at least till a certain age, retain some functional cells at the level of the eyes and respond to light stimuli [72,73], they are blind and use magnetic orientation, thigmotactic stimuli, and chemical cues to move and hunt [74,75,76,77,78,79]. The observed patterns of spring utilization by the olm suggest that the exploitation of surficial habitats by this salamander varied temporally and among different sites. These results highlight the importance of comparing the activity levels of *P. anguinus* between springs and caves to better understand ecological dynamics and the potential influence of seasonal fluctuations. Additionally, there is a clear need of capture–mark–recapture studies to assess site fidelity and movement patterns across adjacent spring habitats, providing a more comprehensive understanding of habitat use and individuals’ dispersal within these more or less interconnected ecosystems. Our study provides evidence that estavelle typology of springs seems to be particularly favorable for the exploitation by *P. anguinus*, especially when they are not connected to sites supporting pikes and other predatory fish. The olms’ preference for temporary springs confirms previous studies performed in the same area, albeit over shorter period of investigation and considering some different abiotic factors [56]. The preferential use by olms of the estavelles with limited maximum water flow is likely related to a reduced drift risk. This finding further indicates that the use of surface habitats by *P. anguinus* is non-random and cannot be solely attributed to individuals observed during a single groundwater flushing event [54]. It is therefore likely that *P. anguinus* engages in a targeted selection of ecotonal environments under conditions that are relatively stable and low-risk. In particular, the avoidance by this species of perennial sites with macrophytes and pikes is potentially promising for further studies assessing the role of Landscape of Fear (LOF) and Hunters’ Horizon (HuHo) frameworks (such as the spatial and temporal variation in predators and prey perception, respectively) in allowing ecotones exploitation [80,81,82,83,84]. These concepts have been developed for endothermic predators and ectothermic surface predators of varying sizes, which must optimize their activity patterns to efficiently capture prey. This involves selecting appropriate strategies, targeting optimal resource sizes (e.g., many small prey versus few large prey, energy-rich versus energy-scarce prey), and meeting high energetic requirements [85,86,87]. As an ectotherm with a slow metabolism adapted to subterranean habitats lacking food resources, the olm can be a valid model to study different processes affecting foraging activity. The olm’s utilization of patches in ecotones at the border with groundwater can provide unique opportunities for testing the limits of marginal value theory and other aspects linked to foraging strategies.

The other species regularly linked to estavelles across the different seasons were two frogs with distinct ecological requirements: *Rana latastei*, an explosive-breeder typical of lowland forest habitats and of conservation interest, and *Pelophylax* sp., a long breeder typical of marshes, ponds, and various lentic aquatic environments that is still relatively common and widespread. *R. latastei* is typically a stenohygrous species, considered a relic of Po Valley lowland woods with abundant brushwood [88,89]. *R. latastei* is predominantly terrestrial outside the breeding season and exhibits seasonal activity patterns, being mainly nocturnal, with activity periods shifting between diurnal and crepuscular. It is primarily active during wet conditions but can also be active in dry periods if the substrate maintains sufficient relative humidity [88,90,91,92]. Females typically select perennial sites for oviposition, such as ponds, pools, small lakes, retting pits, flood pools, fossil bights of watercourses, canals, and ditches [61,91,93]. They usually prefer larger, deeper sites with abundant submerged branches and a certain level of forest cover in the surrounding landscape [61,94,95] where the breeding success can be negatively affected by different invasive predators, especially the red swamp crayfish *Procambarus clarkii* [96,97,98]. Our results suggest that the species is quite spread out in the study area. In estavelles, higher individual counts during winter and autumn were observed in this study, indicating that these sites are utilized outside the breeding season, likely for shelter and foraging in wet conditions. Previous studies have also documented spring use beyond reproduction by the Italian agile frog, particularly in specific spring types such as draining galleries [99]. Draining galleries, like estavelles, appear especially important during the dry periods of summer and late spring, providing critical subterranean refuge. During our observations, Italian agile frogs often hid quickly in crevices and holes when disturbed, suggesting their use as a shelter. RDA results suggest that the species is linked to springs with high microhabitat heterogeneity at the level of substrate. This can favor such sheltering use of estavelles. However, they likely also use springs for feeding, possibly making displacements in and out of the surrounding forest, especially when the latter is dry. In the past, some authors reported already that in early autumn it is frequent to observe the species near the future breeding sites, sometimes even in water [90,100], especially in the case of males. This pattern seems even more pronounced in the ongoing scenario of global warming with possibilities of unprecedented autumn breeding events [95]. The higher number of individuals recorded in December and January months may reflect displacement toward the main damp areas of the poljes on which estavelles exist, where breeding, or attempts to breed, occurs at these sites, despite the rare observation of calling males.

All observed green frogs appear to belong to *Pelophylax* kl. *esculentus*, though not all individuals were captured and examined in detail. These frogs are primarily aquatic, inhabiting diverse wetlands and water bodies—including temporary and permanent pools, ponds, lakes, swamps, marshes, rice paddies, slow-flowing river and stream sections, ditches, canals, sluices, cisterns, drinking troughs, fountains, retting-pits, and garden tubs [60,101,102,103]. Their breeding season begins between March-April and May-June, varying with altitude, and continues until mid-summer with decreasing intensity [104]. The results of the RDA suggest that adult green frogs prefer to exploit ephemeral springs with standing water. Considering the months in which we recorded the occurrences of amphibians in our study case, it appears clear that green frogs used springs and estavelles outside of their breeding period. It is likely that estavelles served as both wet shelters and foraging sites for dispersing sub-adults and adults. The level of exploitation of estavelles by green frogs was less pronounced than for the Italian agile frog and the olm, but still the occurrence of this species was relatively regular in such ecotones.

The common toad occurred in more than 10% of the surveyed springs, even if its observation was limited to the breeding period when the species performs extensive migrations from terrestrial environments to the aquatic breeding sites [105,106]. The species usually breeds in large ponds and waterbodies with fish populations [107,108]. In our study area, males were calling from the main ponds/lakes occurring in the four poljes, especially Doberdò and Pietrarossa where it is likely that breeding occurs. Estavelles are likely used by the toad as something similar to ephemeral “stepping stones” during their migration, as also suggested by the low correlation of *B. bufo* occurrence with the factors investigated. Occurrence of tadpoles in six estavelles was recorded after massive floods of the poljes.

Considering together all the features of the estavelles that we assessed, a clear positive correlation emerges between the presence of perennial water and other variables, such as aquatic macrophyte occurrence and the presence of a top predator like the pike. This finding reinforces the understanding that stable and ephemeral springs can exhibit markedly distinct ecological dynamics [109,110,111] and use by groundwater-dwelling organisms [56].

Particularly noteworthy is the evidence of estavelles and springs being utilized by both fully aquatic stygobionts and semi-aquatic amphibians, which we often observed occurring together during our surveys. This co-occurrence suggests that these habitats serve as critical ecological interfaces, not only facilitating foraging activities but also mediating interactions between different ecological niches. Building on this observation, future research could use these findings as starting points to plan further field observations to explore how predator-prey interactions at ecotones influence adaptive pressures and habitat use. Specifically, mechanisms such as the Landscape of Fear (LOF) and the Hunters’ Horizon (HuHo) are likely relevant in shaping the behaviors of amphibians within spring and estavelle habitats. The LOF describes animals’ perception of spatial and temporal variations in predation risk, which influences their movement and habitat selection [82,112]. Conversely, the concept of HuHo pertains to predators’ perception of prey availability and catchability across space and time [11,113,114]. Although HuHo has received comparatively less attention, it offers valuable insights into predator decision-making processes and prey vulnerability. The interplay between LOF and HuHo mechanisms provides a compelling framework for understanding behavioral strategies under fluctuating risk conditions like those that can occur in estavelles according to the observed variations in use by both aquatic and semi-aquatic amphibians. Despite their significance, LOF and HuHo concepts have been studied predominantly from the prey’s perspective, with less emphasis on how prey variation influences predator behavior—particularly in species such as olms and frogs. Estavelles and spring habitats are ideal sites for investigating these processes, as they represent ecotones situated at the boundary between groundwater and surface water, experiencing contrasting pressures, especially regarding prey availability and predation risk [11]. Our findings reinforce this perspective, indicating that multiple ecotones operate at the level of estavelles. Beyond the fundamental interface between groundwater and surface water, estavelles also constitute a focal merging point connecting aquatic and terrestrial environments. Species such as the Italian agile frog and the green frog appear to play a significant role in mediating biomass exchanges between these two systems

## 5. Conclusions

Overall, the use of estavelles for breeding was very limited for each amphibian species observed. Reproduction likely occurred in nearby damp lentic habitats connected to the investigated springs. Tadpoles and larvae were rarely observed, probably linked to individuals reaching the springs during flooding events that affect the polje. Most sightings involved adults and sub-adults, suggesting these sites are primarily used outside the breeding season for shelter and feeding.

In conclusion, this study provides the first herpetological assessment of estavelle spring habitats in the Classical Karst Region, highlighting their importance as shelters for epigean species and feeding ecotones for stygobiont salamanders. The relative scarcity of predators, the occurrence of water or moisture even during dry periods, the relative microclimatic stability, and slow water flow underscore their ecological relevance, particularly for *P. anguinus* and *R. latastei.*

## Data Availability

The data presented in this study are openly available in FigShare at: https://doi.org/10.6084/m9.figshare.30225997.v1. Exact location of the sites is blinded to preserve olm populations which are often subject to poaching and obsessive photographing sessions.
